# Knowledge, Attitudes and Self-care Practices associated with Glaucoma among Hospital Personnel in a Tertiary Care Center in North India

**DOI:** 10.5005/jp-journals-10008-1116

**Published:** 2012-10-16

**Authors:** Parul Ichhpujani, Shibal Bhartiya, Manisha Kataria, Prateek Topiwala

**Affiliations:** Assistant Professor, Department of Ophthalmology, Glaucoma Facility Government Medical College and Hospital, Sector 32, Chandigarh, India,; Consultant, Department of Ophthalmology, Glaucoma Facility, Fortis Memorial Research Institute, Gurgaon, Haryana, India; Glaucoma Facility, Department of Ophthalmology, Glaucoma Facility Government Medical College and Hospital, Sector 32, Chandigarh, India,; Glaucoma Facility, Department of Ophthalmology, Glaucoma Facility Government Medical College and Hospital, Sector 32, Chandigarh, India,

**Keywords:** Attitude, Awareness, Glaucoma, Intraocular pressure, Knowledge.

## Abstract

**Purpose:** To determine the level of correct knowledge about glaucoma and attitudes toward blindness prevention and treatment and how these factors influence self-care practices among hospital personnel.

**Methods:** In this tertiary hospital based, cross-sectional study, a random sample of 119 staff members including 23 physicians (nonophthalmologists) and 96 nursing staff were administered a self-designed knowledge, attitudes, practice (KAP) questionnaire about glaucoma.

**Results:** All 119 personnel [34 (28.57%) males; 85 (71.42%) females] were aware of glaucoma. Most physicians (80.76%) and nurses (65.26%) understood that glaucoma was associated with a high intraocular pressure and had an effect on the optic nerve. Twenty-four percent of physicians and nurses did not know that it is important for family members of glaucoma patients to be more concerned about getting the disease. As regards ‘treatment priority’ between cataract, glaucoma and diabetic retinopathy; 76.91% physicians and 60% nurses placed glaucoma first. Out of total blindness, stroke or paralysis, cancer, schizophrenia and heart disease, blindness prevention was first priority for 9 (34.60%) physicians and 15 (15.78%) nurses. A recent visit to an eye practitioner (p = 0.012) was a significant predictor of knowledge of glaucoma as a blinding disease.

**Conclusion:** Educating hospital workers on the symptoms of glaucoma and visual impairment can be an important step toward preventive ophthalmic care.

**How to cite this article:** Ichhpujani P, Bhartiya S, Kataria M, Topiwala P. Knowledge, Attitudes and Self-care Practices associated with Glaucoma among Hospital Personnel in a Tertiary Care Center in North India. J Current Glau Prac 2012;6(3):108-112.

## INTRODUCTION

As the world population continues to age, the public health impact of vision loss due to eye diseases will continue to grow and glaucoma would remain as the second or the third most common cause of irreversible blindness in the world.^1^ It has been estimated that almost 90% of glaucoma-related blindness can be prevented with early and proper treatment.^1,2^

It has been seen from various population-based studies that the awareness and knowledge of glaucoma among both rural and urban population is remarkably low, specially in developing countries, which has a negative impact on health seeking behavior.^3-5^ Given that the outreach of the health care system in developing countries remains far from optimal, it is essential that each of the health care providers be educated about glaucoma so as to reach a large sector of the population, which does not have access to a comprehensive eye care center.

Despite its significance, little information is available on knowledge, attitudes and self-care practices associated with glaucoma among hospital workers, with the solitary study in this regard being from Nigeria.^6^

This cross-sectional, questionnaire-based study was carried out to determine the level of knowledge about glaucoma and attitudes toward blindness prevention and treatment, and how these factors influence self-care practices among hospital personnel.

## METHODS

The study was carried out at Government Medical College and Hospital, Chandigarh, a tertiary care hospital, associated with multidisciplinary undergraduate and postgraduate training programs located in an urban area of North India.

A self-designed nine-point questionnaire (Table 1) about glaucoma was administered to a randomly selected group of hospital workers. The same interviewers (PI) assessed each of the respondents in terms of their knowledge, attitudes and self-care practices associated with glaucoma.

Awareness was defined as ‘having heard of glaucoma’. Knowledge was defined as when the subject had some understanding of glaucoma in terms of cause and/or symptoms; high pressure in the eye, damage to the nerve of the eye due to high pressure, damage to retina and the presence of colored haloes and pain in the eye.

Each of the subjects was first questioned whether he or she had heard of the disease entity called glaucoma. Further questions pertaining to knowledge of glaucoma were asked only if the subjects responded in the affirmative and were then asked to explain what they knew of the disease.

**Table 1:** **Table 1:** KAP questionnaire for glaucoma

*Name*		*Age/sex*		*Speciality*	
**Awareness**					
• Are you aware of the disease ‘glaucoma’					
– Yes					
– No					
• If yes, what is your source of information?					
– Health show					
– Print media (newspaper/health magazine book)					
– Internet					
– Relative/friend suffering from glaucoma					
– Ophthalmologist					
– Learn during the course of medical training					
**Knowledge**					
• What is your understanding of ‘glaucoma’?					
– High pressure in the eye					
– Damage to the nerve of the eye due to high pressure					
– Damage to retina					
– Colored haloes and pain in the eye					
• Do you think damage due to glaucoma is reversible?					
– Yes					
– No					
• Arrange the diseases according to ‘treatment priority’. Cataract, glaucoma, diabetic retinopathy?					
• Do you think it is important for family members of glaucoma patients to be more concerned about getting the disease?					
– Yes					
– No					
• Where does blindness prevention come on your priority list? Total blindness, stroke or paralysis, cancer, schizophrenia, heart disease					
**Self-care Practice**					
• When did you last visit an ophthalmologist or an optometrist?					
– <1 year					
– 2 years					
– 3 years or more					
– Never					
– Do not remember					
• If you were diagnosed with glaucoma, how would you go along your treatment course?					
– Visit your ophthalmologist regularly and take suggested treatment (eyedrops) consistently					
– It is not an issue to miss a visit to the ophthalmologist or the eyedrops (frequently)					
– Will prefer getting glaucoma surgery done to avoid the hassle of putting life-long eyedrops?					
• If surgery was the only treatment option available, what would you do?					
– Will promptly go ahead with surgery?					
– Try to defer surgery and continue on eyedrops					
– Take eyedrops and start alternative medicine, such as ayurvedic or homeopathic or yoga					

The questionnaire contained a list of possible responses in terms of high pressure in the eye, damage to the nerve of the eye due to high pressure, damage to retina and symptoms of colored haloes and pain in the eye.

The interviewer marked the response provided by the subject against the response it most closely approached on the questionnaire. In case the response did not correlate with any of the responses listed on the questionnaire, it was documented in greater detail.

Self-care practices were ascertained by a set of three questions on their attitudes toward the prevention and treatment of blindness of glaucoma.

All participants were asked to provide an estimate of their last visit to an eye practitioner that is, either an ophthalmologist or optometrist, and the duration since last visit was documented as <1, 2 or 3 years or more years, or never.

### Data Analysis

During statistical analysis, the participants were categorized as either nurses or doctors. Data was analyzed using the SPSS version 16.00 (Chicago, Illinois, USA).

The relationship between awareness of glaucoma and demographic factors, such as age, gender and education status was assessed using the Chi-square test. A two-tailed p-value of less than 0.05 was considered statistically significant.

## RESULTS

Of the 119 hospital personnel (96 staff nurses; 23 non-ophthalmologist physicians) who consented to participate in the study, and answered the questionnaire, 85 (71.42%) were females and 34 (28.57%) males. Overall, 96 (80.7%) subjects had secondary level of education while 23 (19.3%) subjects had tertiary level of education.

### Awareness

All 119 subjects were aware of glaucoma. The sources of information about glaucoma included health shows in 18 (15.1%), print media in 21 (17.6%) and the internet in 17 (14.3%) subjects. A relative/friend suffering from glaucoma was the source of knowledge for 15 (12.6%), while discussions with attending ophthalmologist and learning during the course of study (nursing/medical) accounted for 22 (18.5%) and 94 (78.9%) subjects, respectively. As is evident from the distribution, several subjects reported more than one source of information about the disease process.

### Knowledge

Most physicians (80.76%) and nurses (65.26%) understood that glaucoma was associated with a high pressure and had an effect on the optic nerve (Table 2).

Twenty-four percent of physicians and nurses did not know that it is important for family members of glaucoma patients to be more concerned about getting the disease. Forty-seven (47,39.5%) subjects thought that the damage due to glaucoma is reversible.

As regards ‘treatment priority’ between cataract, glaucoma and diabetic retinopathy; 76.91% physicians and 60% nurses placed glaucoma first. Out of total blindness, stroke or paralysis, cancer, schizophrenia and heart disease, blindness prevention was first priority for 26 subjects (21.8%) [9 (34.6%) physicians; 15 (15.78%) nurses].

The only significant predictor of knowledge of glaucoma as a blinding disease were recent visit to an ophthalmologist (p = 0.001); while medical profession (p = 0.227) and female sex (p = 0.482) were not found to be significantly predictive of knowledge of the disease.

### Self-care Practices

Only 50 (42%) subjects had visited an ophthalmologist within the past year. Overall, 92 (77.3%) subjects answered that if they were diagnosed with glaucoma, they would visit their ophthalmologist regularly and take suggested treatment (eyedrops) consistently. If surgery was the only treatment option available then 103 (86.6%) subjects were willing to promptly go ahead with the surgery (Table 3).

**Table 2:** **Table 2:** Understanding of glaucoma

* Understanding of glaucoma*		*(n = 119)*	
High pressure in the eye		80 (67.2%)	
Damage to nerve due to high pressure		56 (47.1%)	
Damage to retina		26 (21.8%)	
Colored halos and pain in the eye (Some subjects gave more than 1 response)		24 (20.2%)	

**Table 3:** **Table 3:** Self-care practice along treatment course of glaucoma

*Practice along treatment course of glaucoma*		*(n = 119)*	
Visit your ophthalmologist regularly and take suggested treatment (eyedrops) consistently		92 (77.3%)	
Not an issue to miss a visit to the ophthalmologist or frequently miss a dose of medication		8 (6.7%)	
Will prefer getting glaucoma surgery done to avoid the hassle of putting lifelong eyedrops?		18 (15.1%)	
If surgery was the only treatment option available?			
Will promptly go ahead with surgery?		103 (86.6%)	
Try to defer surgery and continue on eyedrops		10 (8.4%)	
Eyedrops; start adjunctive alternative medicine		4 (3.4%)	

## DISCUSSION

Promoting awareness of common eye diseases and implementing health care programs in a given community can bring forth people to have an eye examination.^3,7^ In a disease like glaucoma, early diagnosis and institution of treatment can result in reduction of visual impairment and blindness, since the major predictor of eventual blindness is late presentation.

Various studies have emphasized a poor awareness of eye diseases among the general population,^4,5,7,8^ with approximately 50% of patients with glaucoma being unaware of their condition at the time of diagnosis^9^ and present in the advanced stage of the disease.^10,11^

It also must be kept in context that there have been few studies demonstrating the association of late presentation of glaucoma with social factors from the United Kingdom^12-14^ and India^3^ where glaucoma is a significant cause of blindness. It was observed in Moorefield’s Eye Hospital Study that glaucoma diagnosis was missed by optometrists and sometimes, ophthalmologists, perhaps because a comprehensive eye examination was not performed.^12,13^ In fact, analysis of late attenders in the clinic revealed that people referred by any source other than an optometrist who has made the correct diagnosis of glaucoma were 4.5 times more likely to be late attenders than patients, so referred but similar in other mentioned factors. Similar findings were observed in the Barbados eye studies where visits to the optometrists still left many patients unaware about their glaucomatous condition.^15^ It is therefore imperative that hospital staff be aware of glaucoma and its implications in order to improve case finding and initiation of treatment at an early stage.

In this study, a startling fact that emerged was that as many as 20% doctors and 35% nurses working in a tertairy care center affiliated to a university teaching institution did not understand that glaucoma was associated with a high pressure and had an effect on the optic nerve. Almost 1 in 4 doctors and nurses did not realize that it is important to screen family members of glaucoma patients for the disease, since they have a higher-risk of disease. Forty percent of those interviewed actually believed that the damage due to glaucoma is reversible.

The implications of these findings are that as many as one in four nonophthalmologists and nurses may not be considered as potential sources of knowledge for patients who reach a tertiary care hospital, and in fact, if questioned, may mislead these patients.

In a similar study among doctors, nurses and non-medical hospital staff from the Obafemi Awolowo University Teaching Hospital in Nigeria^6^ revealed equally disheartening results.

Almost one in two (48.8%) subjects consisting of 38 doctors, 53 nurses and 9 nonmedical staff did not know whether visual loss due to glaucoma was permanent or reversible. Unlike our study, female gender (p = 0.003), secondary or tertiary level of education (p = 0.001), were found to be significant predictors of knowledge of glaucoma as a blinding disease. Like in our study, a recent visit to an eye practitioner (p = 0.012 in Nigeria *vs* 0.001 in our study) was a significant predictor of knowledge of glaucoma.

Almost half the subjects in Nigeria believed that treatment of glaucoma should be given highest priority compared with other diseases, as against 61% in our study (73/119; 76.9% physicians and 60% nurses).

It must also be kept in mind that more than 20% of the subjects (2 doctors and 22 nurses) did not cite their medical education as the source of information about glaucoma and its implications. It must therefore be considered as a serious lacuna in the design of the education curriculum for, both, doctors and nurses.

Despite working in a hospital, with easy and free access to health care, only 42% of the subjects had visited an ophthalmologist within the past year. Barriers to seeking health care, therefore, are more than just access to affordable health care and knowledge about the disease and its implications.

Reluctance to regular visits to the ophthalmologist and the use of medication (eyedrops) was reported by more than 20% of subjects in case of a probable diagnosis with glaucoma.

In contrast, less than 15% reported a hesitation, if surgery was offered to them as the only treatment option available.

## CONCLUSION

Given that the doctors and nurses in departments other than ophthalmology are often the first point of contact when patients seek medical advice, these health care providers must be well-informed or the risk of misinformation and wrong counseling is very high, even in a tertiary care center. It must be kept in mind that it is incorrect to assume adequate knowledge and appropriate attitude among nonophthal-mologists and nurses, as regards glaucoma.

It is, therefore, important to emphasize on intensive eye health education and information dissemination, especially among health care professionals. Educating hospital personnel about the presentation and outcome of this ‘silent thief of sight’ can be an important step toward preventive ophthalmic care. Continuing medical education about glaucoma symptoms and subsequent visual impairment, therefore, must be a priority when designing programs for community outreach.
